# Spectrophotometric method for simultaneous estimation of atenolol in combination with losartan potassium and hydrochlorothiazide in bulk and tablet formulation

**DOI:** 10.4103/0975-7406.72144

**Published:** 2010

**Authors:** Sanjay Bari, Shital Sathe, Pritam Jain, Sanjay Surana

**Affiliations:** Department of Pharmaceutical Chemistry, R. C. Patel Institute of Pharmaceutical Education and Research, Shirpur, Dhule (M.S.) - 425 405, India

**Keywords:** Atenolol, hydrochlorothiazide, losartan potassium, spectrophotometric method

## Abstract

**Aim:**

To develop a simple, accurate, rapid and precise UV spectrophotometric method for the estimation of atenolol in combination with losartan potassium and hydrochlorothiazide.

**Materials and Methods:**

The method employs formation and solving simultaneous equation using 251.60 nm and 224.20 nm for losartan potassium and atenolol, 224.20 and 271.60 for atenolol and hydrochlorothiazide as two analytical wavelengths, using methanol water as a solvent.

**Results and Conclusion:**

The linearity was observed in the concentration range of 5-30 *µ*g/ml (r=0.9991) for losratan pottassium, 2 - 12 *µ*g/ml (r = 0.9995) for atenolol and 2 - 14 *µ*g/ml (r = 0.9993) for hydrochlorothiazide. The results of the method were validated statistically and by recovery studies.

Losartan potassium (LOK) is an angiotensin II receptor antagonist and chemically it is 2-n-butyl-4-chloro-5-hydroxymethyl-1-[2’-(1H-tetrazol-5-yl)(biphenyl-4-yl)methyl]imidazole, a strong antihypertensive agent.[1] Atenolol (ATL) is a cardioselective β-blocker and chemically it is (RS)-4-(2 hydroxy-3-isopropylaminopropoxy)-phenylacetamide.[2,3] Hydrochlorothiazide (HCTZ) is chemically 6-chloro-3, 4-dihydro-2H-1, 2, 4-benzothiadiazine-7-sulfonamide1, 1-dioxide. Literature survey revealed that visible spectrophotometric,[4,5] HPLC[6] and some spectrophotometric,[7,8] HPLC[9] methods are available for the estimation of LOK and ATL from pharmaceutical formulations respectively. Also, different methods are available for the estimation of HCTZ.[10–13] So it was thought to develop an analytical method for the determination of all three drugs in their combined dosage form.

In the present investigation, an attempt has been made to develop a simple and economical spectrophotometric method with greater precision, accuracy and sensitivity for the analysis of atenolol, losartan potassium and hydrochlorothiazide in bulk and dosage forms.

## Experimental

### Instrumentation

The present work was carried out on Schimadzu UV-2450 series spectrophotometer having double beam detector configuration. The absorption spectra of reference and test solutions were carried out in 1 cm quartz cell over the range of 200 - 400 nm.

### Solvents

Methanol (AR Grade, S.D. Fine chemicals, Mumbai, India) and distilled water.

## Procedure: Employing simultaneous equations (Shimadzu 2450)

**Figure 1:**
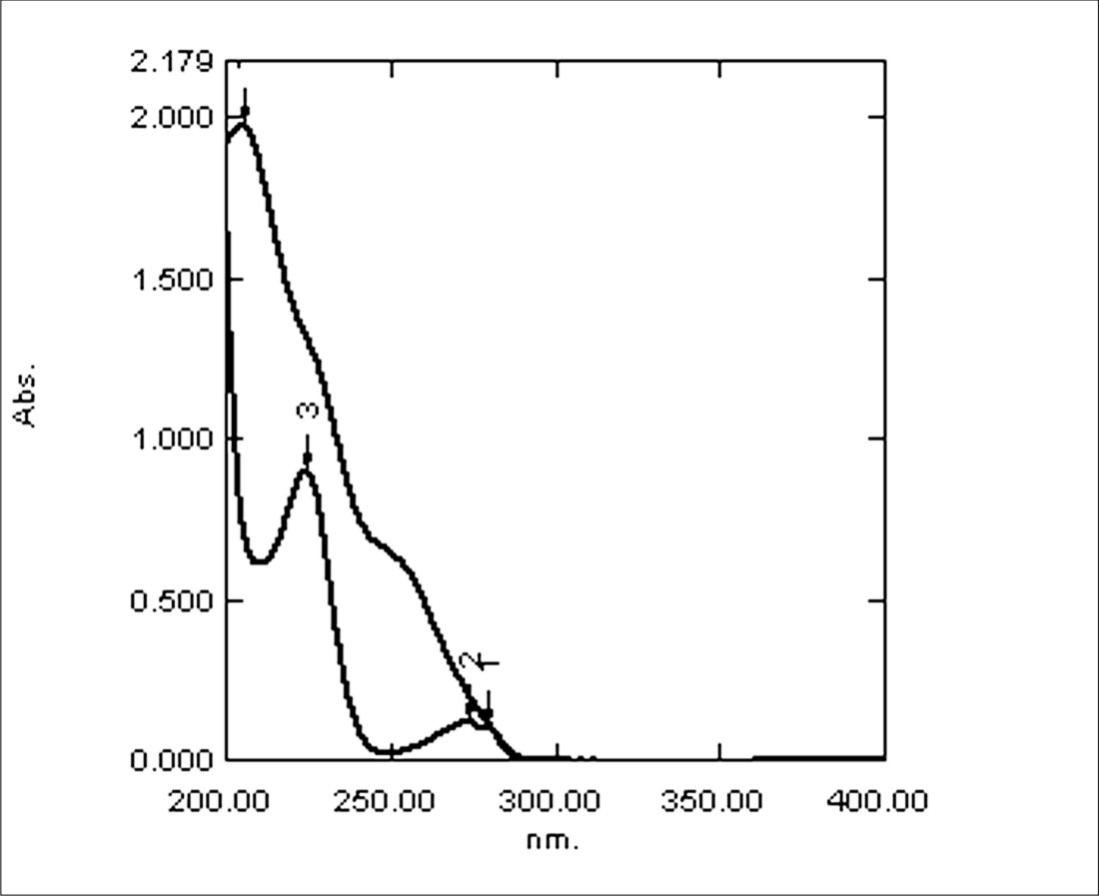
Overlain spectra of LOK and ATL in methanol water

**Figure 2:**
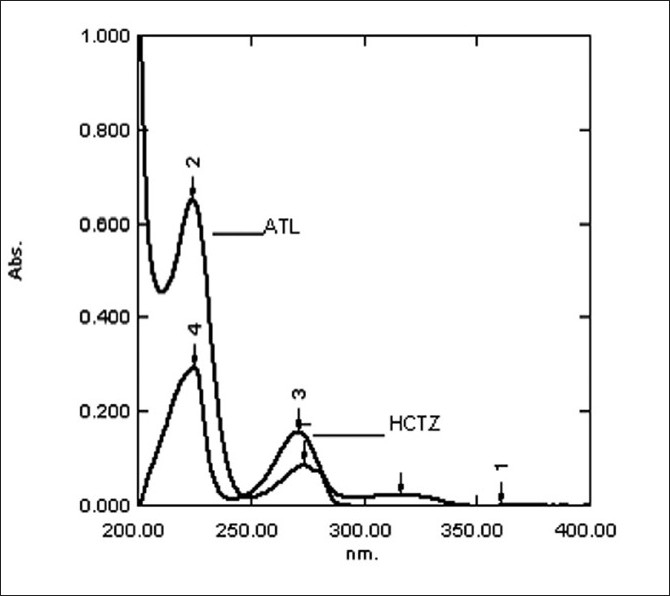
Overlain spectra of ATL and HCTZ in methanol water

Standard stock solutions of LOK, ATL and HCTZ were prepared separately by dissolving 10 mg of each drug in 20 ml methanol and diluted to 100 ml with distilled water. From the overlain spectra (shown in Figure 1) of LOK (20*µ*g/ ml) and ATL (20 *µ*g/ml), two wavelengths 251.60 nm of LOK and 224.20 nm of ATL were selected. The overlain spectra (shown in Figure 2) for ATL (8*µ*g/ ml) and HCTZ (2*µ*g/ ml), two wavelength 224.20 nm and 271.60 nm, were selected respectively. The linearity was found to be in the range of 5 - 35 *µ*g/ml, 2 - 12 *µ*g/ml and 2-14*µ*g/ ml for LOK, ATL and HCTZ, respectively. The optical characteristics and statistical data of regression equation are shown in Table 1.

**Table 1:** Optical characteristics and statistical data of the regression equation

Parameters	LOK	ATL	HCTZ
λ_max_	251.60 nm	224.20nm	271.60nm
**Beer’s law limit (*µ*g/ml)**	5-30	2-12	2-14
**Slope**	0.03315	0.08503	0.06673
Y -intercept	0.02127	0.01713	0.00657
Correlation coefficient	0.9991	0.9995	0.9993

The method is based on simultaneous equation[11] and utilizes corresponding absorbance maximas, i.e. 251.60 nm of LOK, 224.20 nm of ATL and 271.60 nm for quantification. The mean absorptivity coefficients of both drugs at each wavelength were determined from different dilutions (six independent determinations) of corresponding drugs within Beer’s law concentration range limit. Using these, a set of two simultaneous equations were framed for two combinations:

Simultaneous equation for LOK and ATL

(1) A251.60 = C320.25LOK + 8.1 CATL...

(2) $$A_{224.20}\;=\;661.75\;C_{\mathrm{LOK}}\;+\;384.50\;C_{\mathrm{ATL}}$$

where, C_LOK_ and C_ATL_ are the concentrations in g /100ml in sample solution.

By rearranging equations 1 and 2, concentration C_LOK_ and C_ATL_ can be obtained as,

(3) $$C_{\mathrm{LOK}}\;=\;\mathrm{A2}\;\times \;8.1\;-\;\mathrm{A1}\;\times \;380.50/-116494.95$$

(4) $$C_{\mathrm{ATL}}\;=\;\mathrm{A1}\;\times \;661.75\;-\;\mathrm{A2}\;\times \;320.25/-116494.95$$

Simultaneous equation for ATL and HCTZ

(5) A224.20 = C866.75LOK + 1288 CATL

(6) $$A_{271.60}\;=\;106.0\;\mathrm{CLOK}\;+\;720\;C_{\mathrm{ATL}}$$

where C_ATL_ and C_HCZT_ are the concentrations in g /100 ml in sample solution.

By rearranging equations 1 and 2, concentration C_LOK_ and C_ATL_ can be obtained as,

(7) $$C_{\mathrm{ATL}}\;=\;\mathrm{A2}\;\times \;1288\;-\;\mathrm{A1}\;\times \;720/-487532$$

(8) $$C_{\mathrm{HCZT}}\;=\;\mathrm{A1}\;\times \;106.0\;-\;\mathrm{A2}\;\times \;866.75/-487532$$

## Preparation and analysis of tablet formulations

Commercial tablets procured from local market were used for analysis. Twenty tablets (Revas AT) were weighed and crushed to obtain a fine powder. An accurately weighed, sample equivalent to 50 mg of LOK was taken in a stoppered volumetric flask (100.0 ml); 20ml of methanol was added and sonicated for 10 min. The solution was filtered through Whatmann filter paper (No. 41) and volume was made up to the mark with distilled water. After appropriate dilutions, the absorbances of the sample solutions were recorded at 251.60 nm and 224.20 nm i.e.A_251.60_ and A_224.20_ equations 3 and 4. Also, the same procedure is repeated for ATEN-H. After appropriate dilutions, the absorbances of the sample solutions were recorded at 224.20 nm and 271.60 nm i.e.A_224.20_ and A_271.60_ equations 5 and 6. The analysis procedure was repeated five times, with tablet formulations of two brands. The results of analysis of tablet formulations are presented in Table 2.

**Table 2:** Results of analysis of pharmaceutical formulations

	Brand I		Brand II	
	LOK	ATL	ATL	HCTZ
Label claimed (mg/tab)	50	50	50	12.5
*Amount found(mg/tab)	49.78	49.61	49.85	12.45
Standard deviation	0.5366	0.8663	0.901	0.5694
% RSD	1.07	1.62	1.81	1.15

* Mean of five estimations

## Recovery studies

The recovery studies were carried out by adding a known amount of standard solution of LOK and ATL to preanalyzed tablet solutions. The recovery studies were carried at 80%, 100% and 120% level. The results of recovery studies are shown in Tables 3 and 4.

**Table 3:** Results of recovery studies of ATL and LOK

Initial amount (µg/ml)		Concentration of excess drug added to analyte (µg/ml)		% Recovery n = 3		% RSD	
LOK	ATL	LOK	ATL	LOK	ATL	LOK	ATL
20	20	16	16	101.2	99.46	0.68	1.25
20	20	20	20	100.7	99.81	0.91	1.27
20	20	24	24	100.3	100.19	0.64	0.94

**Table 4:** Results of recovery studies of ATL and HCTZ

Initial amount (µg/ml)		Concentration of excess drug added to analyte (µg/ml)		% Recovery n = 3		% RSD	
ATL	HCZT	ATL	HCZT	ATL	HCZT	ATL	HCZT
8	2	6.4	1.6	100.58	99.93	0.59	1.32
8	2	8	2	99.85	100.57	0.52	0.507
8	2	9.6	2.4	99.58	100.67	0.48	1.16

## Results and Discussion

The proposed method for the determination of atenolol, linear regression of absorbance on concentration gave the equation Y= 0.01713X + 0.08503 with a correlation coefficient (r) of 0.9995; for losartan potassium, linear regression of absorbance on concentration gave the equation Y= 0.02127X + 0.03315 with a correlation coefficient (r) of 0.9991; and for hydrochlorothiazide, linear regression of absorbance on concentration gave the equation Y= 0.00657X + 0.06673 with a correlation coefficient (r) of 0.9993. Recovery studies were carried out at three different levels, by adding 80%, 100% and 120 % of pure drug solution to different samples of tablet powder solution. From the amount of drug found, percentage recovery was calculated. Precision was calculated as repeatability (% RSD) and inter and intra day variation (% RSD) for both the drugs. The repeatability and ruggedness data are presented in Table 5. Both the methods were successfully used to estimate the amount of ATL in combination with LOK and HCTZ present in the marketed formulation.

**Table 5:** Repeatability and ruggedness data

Parameters	Method 1		Method 2	
	LOK	ATL	ATL	HCTZ
Repeatability (%RSD (n=5)	0.89	0.31	0.72	0.29
Precision (%RSD)				
Intra-day (n=3)	0.27 - 0.082	0.14 - 0.052	0.17 - 0.052	0.24 - 0.063
Inter-day (n=3)	0.25 - 0.075	0.12 - 0.035	0.14 - 0.068	0.18 - 0.065
Ruggedness (%RSD)				
Analyst 1	1.59	0.79	1.25	0.71
Analyst 2	1.21	0.72	1.18	0.68

## Conclusion

Atenolol exhibited maximum absorption at 224 nm and obeyed Beer’s law in the concentration range of 2-12 *µ*g / ml; losartan potassium exhibited maximum absorption at 251 nm and obeyed Beer’s law in the concentration range of 5-30 *µ*g / ml; and hydrochlorthiazide exhibited maximum absorption at 271 nm and obeyed Beer’s law in the concentration range of 2-14 *µ*g / ml. The percentage recovery value for atenolol was 99.46% to 100.58%; for losartan potassium was 100.3% to 101.2 %; and for hydrochlorthiazide was 99.93% to 100.67%. This indicates that there is no interference of the excipients present in the formulations. The developed method was found to be accurate, precise, repeatable and reproducible and can be used for the routine analysis of atenolol, losartan potassium and hydrochlorthiazide in bulk drug and formulations.
